# Supplementary material for: Modeling the impact of adherence to U.S. isolation and masking guidance on SARS-CoV-2 transmission in office workplaces in 2021–2022
Source: Epidemics. Author manuscript; Available in PMC 2026 Sep 30. (PMC13624584; doi:10.1016/j.epidem.2026.100935)
Supplement: Supplementary file 1 [file NIHMS2210439-supplement-Appendix1.pdf]

Supplementary Methods: Model Specification

The model was implemented in R using the EpiModel software platform (Jenness SM, Goodreau SM, Morris M. EpiModel: An R Package for Mathematical Modeling of Infectious Disease over Networks. J Stat Softw. 2018;84(8):1–47), which uses exponential random graph models (ERGMs) to estimate and simulate dynamic contact networks. The present model extends EpiModel through EpiModelCOVID, a purpose-built COVID-19 module that implements the SARS-CoV-2-specific disease-state structure, vaccination logic, diagnostic testing, and CDC isolation and masking guidance pathways described in the sections below. Full model code and software are publicly available at https://github.com/EpiModel/COVID-Corporate.

S1. Disease-State Structure

The model uses a Susceptible–Exposed–Infectious–Recovered–Susceptible (SEIRS) framework extended to distinguish four infectious sub-states. Each agent is assigned exactly one of the seven disease states listed in Table S-1 at any given time step (day). Each agent's assigned disease state is stored in their `status` attribute. Transitions between states are discrete-time, stochastic, and evaluated at each daily time step.

Table S-1. Disease states

Status code	Name	Description	Infectious?
**S**	Susceptible	No immunity; eligible for infection	No
**E**	Exposed (latent)	Infected but not yet infectious; asymptomatic	No
**Ip**	Pre-clinical	Infectious; no symptoms yet; destined for clinical course	Yes
**Ic**	Clinical (symptomatic)	Infectious and symptomatic; may be hospitalized	Yes
**H**	Hospitalized	Severe illness; contacts assumed effectively zero	Yes*
**A**	Asymptomatic/subclinical	Infectious; will not develop symptoms	Yes
**R**	Recovered	Temporarily immune; immunity wanes stochastically	No

** Hospitalized agents are assumed to have no effective contacts for transmission purposes.*

Upon infection (S → E) an agent is probabilistically assigned to the clinical pathway (destined for symptomatic Ic) or the subclinical pathway (destined for asymptomatic A) using an age- and vaccination-adjusted Bernoulli draw (Section S4). Clinical-pathway agents traverse E → Ip → Ic → {H or R}; subclinical-pathway agents traverse E → A → R. All recovered agents re-enter susceptibility stochastically as immunity wanes.

S2. State-Transition Rates

At each time step, transitions out of each state are evaluated as independent Bernoulli draws with daily probability r = 1/D, where D is the assumed mean duration in days. Table S-2 lists each transition, the parameters governing it, and the mean duration used.

Table S-2. State transitions and daily rate parameters

Transition	Rate parameter	Mean duration (days)	Notes
**E → A**	ea.rate	5.5	Subclinical pathway only
**E → Ip**	eip.rate	5.5	Clinical pathway only

Transition	Rate parameter	Mean duration (days)	Notes
**Ip → Ic**	ipic.rate	1.5	Pre-clinical infectious period
**Ic → H**	ich.rate	3.0	Proportion hospitalized drawn at Ic entry (Section S4)
**Ic → R**	icr.rate	3.5	Non-hospitalized clinical cases
**A → R**	ar.rate	5.0	Subclinical infectious period
**H → R**	hr.rate	15.5	Hospital stay duration
**R → S**	rs.rate	300	Mean natural immunity duration; waning is geometric

S3. Per-Contact Transmission Probability

S3.1 Base probability and layer-specific act rates

For each discordant dyad (susceptible agent i, infectious agent j) active on network layer l ∈ {workplace, community, household} at time step t, the probability that a transmission event occurs during the daily time step is:

$$P_{transmit}(i,j,l,t) = 1 - (1 - q_{ij(l,t)})^{c_{l(t)}}$$

(Eq. 1)

where $c_{l(t)}$ is the layer-specific daily act rate (mean number of contacts per day within layer l) and $q_{ij(l,t)}$ is the per-contact transmission probability, defined as a product of multiplicative modifiers applied to the calibrated probability β:

$$q_{ij(l,t)} = \beta(l,t) \cdot RR_{vax(i,t)} \cdot RR_{asymp(j)} \cdot \alpha_{mask(j,t)}$$

(Eq. 2)

Each factor is described in detail in the following sections. Table S-3 summarizes all modifier parameter values.

Table S-3. Transmission probability modifier parameters

Modifier	Symbol	Value	Reference / source
Reference per-contact transmission probability	β_0	0.38	Calibrated
Relative risk of infection, dose 1	RR_1	0.324	Pilishvili et al., 2021
Relative risk of infection, dose 2	RR_2	0.112	Barda et al., 2021
Relative risk of infection, dose 3	RR_3	0.120	Barda et al., 2021
Relative risk of infection, dose 4	RR_4	0.120	Barda et al., 2021
Vaccine immunity half-life	$\tau½$	80 days	CDC Science Brief, 2021
Relative transmissibility, asymptomatic/pre-clinical infector	RR_{asymp}	0.5	Davies et al., 2020
Relative contact intensity during isolation	α_{iso}	0.3	Assumed
Relative per-contact transmission probability with masking	α_{mask}	0.3	Assumed

S3.2 Reference transmission probability and time-varying calibration modifiers

The reference per-contact transmission probability β_0 = 0.38 was calibrated to reproduce observed monthly infection incidence in Georgia. To account for SARS-CoV-2 variant emergence and shifts in population immunity over the simulation period, β was adjusted by additive increments applied during specified calendar windows (Table S-4). Outside these windows $\beta = \beta_0$. Modifiers are identical across all network layers; no layer-specific baseline transmission probability was fitted.

Table S-4. Time-varying calibration adjustments to the reference transmission probability β_0 = 0.38

Type	Calendar window	Time step range	Increment	β(t)
Baseline	All other periods		—	0.38
Boost	Nov 2020 – Jan 2021	120 – 195	+0.45	0.83
Suppression	Jan – Jun 2021	210 – 345	−0.22	0.16
Suppression	Sep – Nov 2021	430 – 504	−0.30	0.08
Boost	Nov 2021 – Jan 2022	505 – 576	+0.10	0.48
Suppression	Feb – Apr 2022	577 – 665	−0.23	0.15
Suppression	May – Aug 2022	666 – 788	−0.12	0.26

Calendar dates are derived from simulation time steps, with t = 181 corresponding to January 1, 2021 (epidemic start, following a 180-day burn-in). β(t) values reflect the single active modifier for each window; boost and suppression windows do not overlap.

S3.3 Vaccination modifier

Let $v_i(t) \in \{0, 1, 2, 3, 4\}$ denote the number of vaccine doses received by susceptible agent i at time t, and let $\Delta_i(t) = t - t_{vax,last(i)}$ be the time (in days) since the most recent dose. Vaccine-conferred protection wanes exponentially with half-life τ½ = 80 days (i.e., the RR doubling time). The relative risk modifier is:

$$RR_{vax}(i,t) = min\{RR_d(v_i) \cdot 2^{(\Delta_i(t)/\tau½)}, 1\}$$

(Eq. 3)

where $RR_d(v)$ is the dose-specific relative risk of infection at the time of vaccination (RR_1 = 0.324, RR_2 = 0.112, RR_3 = 0.120, RR_4 = 0.120) and the min{·, 1} truncation prevents the modifier from exceeding 1.0 as immunity decays back to baseline. Unvaccinated agents (v_i = 0) receive RR_{vax} = 1.0.

S3.4 Infector symptom-status modifier

Subclinically infected agents (`status` A) are assumed to transmit with lower efficiency than clinically infected agents (`status` ∈ {Ip, Ic}). The infector modifier is:

$$RR_{asymp}(j) = \{0.5 \ if \ status_j = A; \quad 1.0 \ if \ status_j \in \{Ip, Ic\}\}$$

(Eq. 4)

S3.5 Masking and isolation modifiers

An agent attribute labeled `isolate` tracks whether each agent adheres to CDC's isolation and masking guidance, and if so, which guidance pathway they are on (see Section S5). Two intervention-related modifiers operate independently on the act rate (contact intensity) and on the

per-contact transmission probability whenever an agent's `isolate` attribute is non-missing (i.e., the agent is on any guidance pathway).

Isolation (act-rate reduction). If infector j has `isolate` ∈ {1, 2} (active isolation, mild or severe disease), the daily act rate for that dyad is multiplied by the relative-contact-intensity parameter α_{iso} = 0.3. This reduces the expected number of contacts per day from c_l to 0.3 · c_l for that agent in all network layers.

Masking (transmission-probability reduction). If infector j has `isolate` ∈ {1, 2, 3, 4} (any guidance state), the per-contact transmission probability is multiplied by α_{mask} = 0.3 as shown in Eq. 2. Masking acted as source control, reducing the transmissibility of an infected agent but not the susceptibility of a susceptible agent.

Combining these, the effective per-dyad final probability when both modifiers apply is:

$$P_{transmit}(i,j,l,t) = 1 - (1 - q_{ij})^{(c_l \cdot \alpha_{iso})}$$

(Eq. 5)

S4. Vaccination and Waning Immunity in Disease Progression

Vaccine modifiers alter not only the probability of infection (Section S3.3) but also the probabilities of following the clinical infection pathway (i.e., experiencing symptoms), assigned when an agent first becomes exposed (E), and of becoming hospitalized, assigned when an agent first becomes clinically infected (Ic).

Clinical probability. At the E → {Ip or A} branch point, the age-specific proportion symptomatic (prop.clinical[a]) is multiplied by dose-specific relative risks $RR_{clin,d}$ (d = 1, …, 4) and the waning factor $2^{(\Delta_i(t)/\tau½)}$, truncated at the unvaccinated baseline:

$$P_{clin}(i) = min\{prop.clinical[a_i] \cdot RR_{clin,d(v_i)} \cdot 2^{(\Delta_i(t)/\tau½)}, prop.clinical[a_i]\}$$

(Eq. 6)

Hospitalization probability. At the Ic → {H or R} branch point, the age-specific hospitalization proportion (prop.hospit[a]) is similarly modified by dose-specific relative risks $RR_{hosp,d}$, waning, and time-varying calibration multipliers applied during variant surge periods:

$$P_{hosp}(i,t) = min\{prop.hospit[a_i] \cdot RR_{hosp,d(v_i)} \cdot 2^{(\Delta_i(t)/\tau½)}, prop.hospit[a_i]\} \cdot M_{hosp}(t)$$

(Eq. 7)

where $M_{hosp}(t)$ is a time-varying calibration multiplier (boost or suppression) applied during specified periods to match observed hospitalization trends.

S5. CDC Isolation & Masking Guidance-Adherence and Isolation-State Logic

S5.1 Adherence to CDC guidance encoding

Each agent carries an integer attribute `isolate` ∈ {NA, 1, 2, 3, 4} encoding its current adherence to CDC's masking and isolation guidance, and the associated behavioral modifications. Each agent also carries an integer attribute `isoTime` encoding the time step for entry into a given guidance pathway. Table S-5 defines each code.

Table S-5. Adherence to CDC guidance (`isolate`) codes

Code	Label	Definition
**1**	Isolation for mild disease	Active isolation of at least 5 days triggered by symptom onset or positive test (no hospitalization). Act-rate multiplied by α_{iso} = 0.3; transmission probability multiplied by α_{mask} = 0.3.
**2**	Isolation for severe (hospitalized) disease	Transition to H upgrades code from 1 → 2. Same act-rate and masking reductions apply, but isolation duration increases to at least 10 days.
**3**	Post-isolation masking	Entered from R after ≥5 days in state 1/2. Isolation ends; masking (α_{mask}) continues through day 10.
**4**	Post-exposure masking	Triggered by contact-notification. Masking only (α_{mask}) for days 0–10; test on day 6.
**NA**	—	Not on guidance pathway.

S5.2 Entry into a CDC guidance pathway

Entry into a CDC guidance pathway can be triggered by three routes, each conditional on an agent-level adherence probability draw (`iso.prob.office` for office workers; `iso.prob.other` for non-office workers; reference value 0.58 for both):

1. **Symptom onset.** At the Ip → Ic transition, a Bernoulli(`iso.prob`) draw determines whether the newly symptomatic enters the guidance pathway for COVID-19-like illness. If yes, `isolate` ← 1 and `isoTime` ← t.
2. **Positive test (asymptomatic).** If a non-symptomatic agent tests positive (Section S6), a Bernoulli(`iso.prob`) draw determines whether the agent follows the guidance pathway for a positive SARS-CoV-2 test. If yes, `isolate` ← 1 and `isoTime` ← t.
3. **Exposure notification.** Agents with a recently diagnosed contact may be notified with layer-specific probability `notif.prob[l]` (workplace: 0.5, community: 0.1, household: 0.9). Timing windows: household contacts notified within 1 day of diagnosis; workplace and community contacts within 3 days. Notified agents who pass a Bernoulli(`iso.prob`) adherence draw are assigned `isolate` ← 4 (masking only). `isoTime` is set to either the time of the most recent contact edge (community layer) or the time of the contact's diagnosis (household and workplace layers, where contacts are persistent).

S5.3 Progression through and exit from CDC guidance pathways

Once on a pathway, an agent advances through states according to the following rules, evaluated at each daily time step in the disease-progression module:

Agents with `isolate` = 1 (mild disease isolation) who recover (enter R) and for whom (t − `isoTime`) > 5 transition to `isolate` = 3 (post-isolation masking). Agents with `isolate` = 1 who become hospitalized (enter H) transition to `isolate` = 2 (severe disease isolation). Agents with `isolate` ∈ {2, 3} who are in R and for whom (t − `isoTime`) > 10, and agents with `isolate` = 4 for whom (t − `isoTime`) > 10, exit the pathway: `isolate` ← NA, `isoTime` ← NA.

Agents on post-exposure-masking state (`isolate` = 4) are additionally eligible for a diagnostic test on day 6 after `isoTime` (Section S6). A positive result transitions them to `isolate` = 1 and restarts the full isolation protocol.

S6. Diagnostic Testing

Each time step, three pools of eligible agents are screened for SARS-CoV-2:

1. **Symptomatic, not isolating.** Agents with `status` = Ic and `isolate` = NA are tested at rate `dx.rate.sympt` = 0.1.
2. **Non-symptomatic, not isolating.** Agents with `status` ∈ {S, E, A, Ip, R} and `isolate` = NA are tested at rate `dx.rate.other` = 0.01.
3. **On isolation pathway.** Agents with `isolate` ∈ {3, 4} (post-isolation masking, or post-exposure masking after day 5) are all tested.

For agents who undergo testing, true positivity depends on disease status: agents with `status` ∈ {A, Ip, Ic} are truly positive; `status` ∈ {S, E, R} are truly negative. Among truly positive agents, the test returns positive with probability `pcr.sens` = 0.80 (diagnostic sensitivity). A positive result updates `dxStatus` ← 2 and `dxTime` ← t; a negative result updates `dxStatus` ← 1.

Agents who test positive and are not already on a guidance pathway are eligible to enter the pathway for a positive SARS-CoV-2 test via a Bernoulli(`iso.prob`) adherence draw, following the same logic described in Section S5.2.

S7. Daily Stochastic Simulation Loop

The simulation operates in discrete daily time steps t = 1, 2, …, T. Each run comprises a 180-day burn-in period (to allow networks, vaccination, and epidemic seeding to stabilize) followed by 608 days of epidemic time corresponding to January 1, 2021 through August 31, 2022, for a total of T = 788 steps. Reported outcomes are extracted from steps 181–788 only. At each step the following modules are executed in sequence:

Table S-6. Ordered simulation modules executed at each time step t

Order	Module (function)	Actions at each time step t
**1**	Aging (aging_covid)	Increment each agent's age by 1/365 years; update age-group and vaccine-eligibility attributes.
**2**	Departures (deaths_covid_corporate)	Apply age-specific all-cause mortality rates; multiply by mort.dis.mult for hospitalized agents. Remove deceased from all network layers.
**3**	Arrivals (arrival_covid_corporate)	Add new agents (births); assign age 0, susceptible status, and household membership.
**4**	Network re-simulation (resim_nets_covid_corporate)	Re-simulate dynamic community-layer edges via ERGM (tergmLite). Household and workplace layers are static and not re-simulated.
**5**	Transmission (infect_covid_corporate)	For each of the three network layers, enumerate discordant (susceptible–infectious) dyads. Compute per-dyad transmission probability incorporating all modifiers (Eq. 1–2). Draw independent Bernoulli outcomes. Update newly infected agents to state E.
**6**	Disease progression (progress_covid)	Evaluate contact-notification masking for eligible exposed agents. Assign clinical/subclinical pathway to newly exposed agents. Advance agents stochastically through all state transitions (Table S-2). Manage CDC guidance adherence state entries, upgrades, and exits.
**7**	Diagnosis (dx_covid)	Stochastically test symptomatic agents (rate dx.rate.sympt), non-symptomatic agents (rate dx.rate.other), and agents on the guidance

Order	Module (function)	Actions at each time step t
		pathway. Apply PCR sensitivity (0.80). Update dxStatus; trigger isolation for newly positive adherent agents.
**8**	Vaccination (vax_covid)	Draw new vaccinees from dose- and age-group-specific eligible pools using calibrated daily rates. Record dose number and time of vaccination; apply vaccine attribute to agent.
**9**	Prevalence (prevalence_covid_corporate)	Tally all agents in each disease state and epidemiological flow counts for tracked outcomes.

All stochastic draws within a given time step use independently seeded random-number streams. The simulation is run for 250 independent realizations per scenario, each initialized with 100,000 agents and 1,600 initially exposed agents (`status` E) seeded at t = 1.

Network layers differ in their persistence. Household edges are fixed throughout the simulation (static). Workplace edges are also persistent (static). Community edges are re-drawn at every time step via simulation from an exponential random graph model (ERGM) using the *tergmLite* engine within EpiModel, producing a new set of community contacts each day while preserving the specified marginal degree distribution and age-assortative mixing.

Within the transmission module, the discordant edgelist – pairs (i, j) where i is susceptible and j is infectious (`status` ∈ {A, Ip, Ic}) – is extracted separately for each layer. Per-dyad probabilities (Eq. 1) are computed and independent Bernoulli draws determine whether each susceptible is infected. If a susceptible agent appears in discordant pairs from multiple layers in the same time step, each exposure is resolved independently; the agent may only be infected once per step (the first drawn positive result takes precedence via the use of `unique()` on newly infected IDs).
